# Taking the path of least resistance: a qualitative analysis of return to work or study while breastfeeding

**DOI:** 10.1186/s13006-019-0209-x

**Published:** 2019-04-04

**Authors:** Elaine Burns, Zoi Triandafilidis

**Affiliations:** School of Nursing and Midwifery, Western Sydney University, Locked Bag 1797, Penrith, New South Wales Australia

**Keywords:** Breastfeeding, Organisational support, Workplace, Student, Qualitative, Thematic analysis

## Abstract

**Background:**

In order to meet World Health Organization recommendations for breastfeeding, many women need to combine breastfeeding with return to work or study. Barriers are often encountered when returning to work or study, which can lead to premature cessation of breastfeeding. This study aimed to explore Australian women’s experiences of breastfeeding at one multi-campus university.

**Method:**

This paper draws on the qualitative findings from a mixed-methods study conducted between April and November 2017. An online survey was used to explore women’s experiences of breastfeeding at university. In total, 108 people participated in the survey. After the deletion of incomplete surveys, 79 staff and students survey responses were analysed. In-depth interviews were also carried out with 10 staff and students. Open text responses and in-depth interviews were analysed using thematic analysis.

**Results:**

The analysis revealed four themes. The first theme, *University as a positive and progressive environment for breastfeeding*, explores staff and students’ experiences of maternity leave, flexible work arrangements, and on-campus childcare, and their relationships with tutors, supervisors, managers and colleagues. The second theme, *Finding private and safe spaces for breastfeeding,* presents staff and students’ experiences of using designated rooms, car parks, corridors, classrooms, and offices to breastfeed and express breast milk, and their experiences related to storage of breast milk. The third theme, *Feeling self-conscious and unprofessional*, reflects women’s experiences of mixing their professional and personal lives, and feeling guilty for taking time out to breastfeed. The fourth theme, *Developing resilience to judgement,* captures women’s realisation that breastfeeding on campus requires the development of a “thick skin” and the capacity to not be offended easily.

**Conclusions:**

Sustaining breastfeeding requires time and commitment on behalf of the mother, as well as a supportive workplace or study environment. Transforming university campuses into breastfeeding friendly environments is long overdue and requires organisational commitment to achieve genuine reform.

## Background

According to the recent Lancet series, breastfeeding makes the world “healthier, smarter and more equal” ([1] p. 404). The World Health Organization [WHO] recommends exclusive breastfeeding up to 6 months of age, with continued breastfeeding, along with appropriate complementary foods, for 2 years of age or beyond [2]. Current data on breastfeeding duration in Australia reveal that many women abandon exclusive breastfeeding before the recommended durations, with only 15% of infants exclusively breastfeeding to 5 months of age [3]. In order to meet the WHO recommendation, mothers will often need to combine breastfeeding with return to work or study.

In high-income nations such as Australia, the rates of breastfeeding, and the duration of breastfeeding, are significantly lower than in poorer countries [4]. This is a worrying statistic given that access to education, and health information, is higher in affluent countries [4]. International data reveals that one of the key factors in women’s decision to cease breastfeeding is return to work or study. Cessation is more prevalent if the return to work or study occurs before the infant is 6 months old [5–10]. There is limited Australian data on breastfeeding rates and return to work or study; however, recommencement of paid employment has been identified as having a significant impact on breastfeeding cessation for women in Australia [3, 11].

Australian workplace gender equality, and anti-discrimination legislation currently protects the rights of women with regard to breastfeeding and return to work [12, 13]. This study aimed to explore women’s experience of returning to work or study whilst maintaining breastfeeding at one higher education facility in Australia. The study sought to identify the experiences, facilitators and barriers around combining return to work or study while breastfeeding.

Existing research has shown that inadequate breastfeeding facilities in the workplace are a risk factor for breastfeeding discontinuation [14]. A cross-sectional survey carried out in New South Wales, Australia, found that women returning to work felt largely unsupported by their managers to continue breastfeeding, and women instead had to rely on support from family and partners to combine breastfeeding and work [11]. Some studies on workplace provisions for breastfeeding women have shown overall positive attitudes amongst employees [15].

There are currently eight universities in Australia that have achieved “Breastfeeding Friendly Workplace” accreditation [16]. This study will focus on one university, which has not yet sought breastfeeding accreditation. The university allocates spaces for all staff and students to breastfeed, or express breast milk, and has provisions for full-time employees to take up to 52 weeks paid maternity leave with paid lactation breaks on return to work. The aim of this study was to explore women’s experiences of combining return to work, or study, while maintaining breastfeeding, at an Australian university. Some authors have explored the impact of employment status on experience of return to work and breastfeeding [17–19], but this is the first Australian study of a higher educational institution that includes the experiences of students.

## Method

### Study aims and design

The aim of this study was to explore women’s experience of combining return to work or study whilst maintaining breastfeeding. The study was designed to meet the following objectives:

1. To determine what facilitators and barriers women experienced in returning to work or study whilst breastfeeding;
2. To explore the day-to-day practicalities of combining work or study and breastfeeding; and
3. To capture how women felt about navigating the university when breastfeeding.

This study utilised a mixed-methods design [20], which involved an online survey of 108 staff and students from a university in Australia during 2017, and in-depth interviews with 10 women. The quantitative data from this survey will be presented in a forthcoming publication. This paper will report on the qualitative components of the study.

The online survey generated 108 responses. Incomplete responses or surveys from participants who did not meet the inclusion criteria were removed by the authors. A total of 79 survey responses remained for analysis. Audio-recorded in-depth interview data were transcribed verbatim by a professional transcription company, prior to analysis. Survey extended response data, and qualitative in-depth interviews, were analysed using thematic analysis. The study design was approved by the Western Sydney University Human Research Ethics Committee H12272.

### Setting

The study was conducted across ten campuses at one university in Australia. This university had three policies that related to parents on campus: Family Responsibilities in the Workplace; Supporting Parent’s Toolkit for staff; and Children on University Premises. The first two policies applied to staff and the latter policy applied to both staff and students.

### Participants

Participants were recruited through a flyer that was distributed across the university campuses and online through social media. Participants were women who were a staff member or a student at the university, and were currently breastfeeding, or had combined breastfeeding and return to work or study in the past 2 years. Participation in the study was voluntary, and all responses were anonymous and confidential.

### Data collection

#### Online survey

The online survey (OLS) asked participants about the provision of breastfeeding friendly spaces at the university. The survey took approximately 15 min to complete and was created and distributed using the online survey platform, Qualtrics.

The online survey also contained some open-response questions such as: *Tell us about your experiences of expressing milk on campus?* or *How might the university better support those breastfeeding on campus?*

#### Interviews

At the end of the online survey, women were asked if they would be interested in participating in an in-depth interview about their experiences of breastfeeding at university. The interviews were semi-structured, including a small number of open-ended questions, and were conducted in a conversational style [21]. The women were asked questions such as, “*What are some of the positive aspects of breastfeeding on campus?”* and “*What are some of the challenges you’ve faced in breastfeeding on campus?”*

Three interviews were conducted face-to-face, and the remaining seven were completed over the telephone. The duration of the interviews ranged from 25 min to an hour. With participants’ consent, the interviews were audio recorded.

#### Analysis

The in-depth interview transcripts were integrity checked by the second author, by listening to the audio recordings, checking for errors, and making corrections where necessary. While integrity checking, the transcripts were anonymised by removing names, places, and identifying information. This process helped the authors to become more familiar with the data prior to analysis [22, 23]. Having familiarised themselves with the data, some of the concepts or common themes became apparent in the data. The interview and online survey data were then imported into NVivo 11, a computer software programme that allows for the compilation, management, and coding of qualitative data.

Following transcription, the data were coded: a process that allowed the authors to organise the data into themes. The process of coding involved identifying both semantic and latent content. Semantic themes operate at an explicit level, on the surface, while latent themes are on an interpretative level, and recognise underlying ideas and concepts [22]. For example, “location of childcare” and “breast milk storage” were codes that captured semantic themes, while “confidence with breastfeeding” and “the importance of privacy” were codes that captured latent meaning. Both authors worked together to refine the coding frame through the renaming, collapsing, removal, and separating out of codes. The development of the coding frame was inductive, as it was based on the data, rather than any pre-existing theoretical or analytic interest [22]. Once all the data had been coded, the content of each code was summarised, and themes were then identified.

## Results

The majority of the 79 survey respondents were born in Australia [66%], had spent one to 5 years at the university [53%], were no longer breastfeeding [63%], and had one [49%] or two [28%] children. The ten interview participants were employed as staff (*n* = 8) or enrolled as students (*n* = 2) at the university. Some of the staff were also, or had also, been students at the university. A maximum variation sample of interview participants were recruited, which included undergraduate and postgraduate students, and staff in professional and academic roles, on casual and permanent contracts.

Thematic analysis of the qualitative survey and interview data revealed four themes: *University as a positive and progressive environment for breastfeeding; Finding private and safe spaces for breastfeeding; Feeling self-conscious and unprofessiona*l; and *Developing resilience to judgement*.

### Theme one: university as a positive and progressive environment for breastfeeding

Many women described the university as a positive environment for breastfeeding. A woman who had been both a student and a staff member at the university said, “The university has made it very easy for me to be a working mum...the facilities and the policies do really support the combination of work and family, or study and family.” Another woman said:

I was so proud to be a staff member and student of an institution that truly 'walked the talk' about being family friendly. I am not sure if my experience is common but it was exceptional. [OLS]

A staff member spoke about the parenting and breastfeeding information given on the university website, saying, “It seemed like it was a supportive environment. I just went, ‘Wow, okay. This place obviously has some structure in there that they’re supportive of this’”. Compared to other working environments, the university was described by staff as progressive, as demonstrated by the following accounts from staff: “in my first job, my manager said, ‘Oh, can’t you do it in the toilet?’”; or the following: “After coming from an organisation [which] requested I express in a storeroom with no power point, I was pleased to know that policies and support existed at [this university]”.

#### Maternity leave, flexible work arrangements, and campus childcare

Staff and students discussed university policies and facilities that assisted them with breastfeeding, which included maternity leave, flexible work arrangements, and campus childcare. Participants who were permanent staff members at the university spoke about the benefits associated with paid maternity leave, saying it “made a big difference”. The capacity to delay return to work until the infant turned 12 months old was seen as a positive step in terms of breastfeeding sustainability. Staff identified that by the time they returned to work they would only need to feed before and after work and could manage the daytime by expressing for comfort if needed.

Staff also spoke about how the ability to be flexible with regards to work assisted with breastfeeding. For instance, a permanent academic and postgraduate student, said, “you can dictate the hours of availability, to a certain extent, that you can teach. That certainly helped...”. Similarly, a professional staff member spoke of the benefits of flexible work arrangements saying:

I have recently started to wean my child off the breast and have been experiencing very painful breast engorgement. My manager has been very supportive and has provided the opportunity to work from home so that I can be more comfortable. [OLS]

Having access to on-campus childcare was identified by some women as a key factor in maintaining ongoing breastfeeding, as the following quote from a professional staff member demonstrates:

The university childcare centres are very supportive and encouraging of breastfeeding. When my child was transitioning into care, she did not take a bottle. The centre would ring me whenever my daughter needed a feed and was refusing a bottle — I would attend the centre, where they would make a comfortable chair available, and I would feed my child. I never felt out of place or uncomfortable. [OLS]

Casual staff members faced additional challenges with breastfeeding as they did not have access to maternity leave and often had shared office spaces. Casual staff were employed on a short-term contract, therefore they could choose whether or not to work during a given semester. However not all casual staff could afford to take a semester off. One casual staff member described having to return to work soon after experiencing a traumatic birth and said her tenuous employment situation had “a significant impact” on her ability to breastfeed. She said:

Being casual means I have no rights . . . two weeks into the start of semester, so two weeks of delivering lectures, I just had to stop breastfeeding. I couldn't do it, because of the stress and being out and not just able to feed . . . my supply went from being pretty shit, to be just abysmal, so I stopped. [IV 6]

#### Having the support of tutors, supervisors, managers, and colleagues

Having the support of tutors, supervisors, managers, and colleagues was an important factor in women’s breastfeeding experiences, as one postgraduate student and casual academic said: “I’ve had a really wonderful opportunity to be surrounded by people who have supported me”. These experiences were reported by undergraduate and postgraduate students and staff, as demonstrated in the following accounts:

I have been very lucky to breastfeed and have positive teachers who have happily allowed me to bring my baby to class. This has had a huge impact on my breastfeeding and parenting experiences these past nine months. [OLS]

I was able to breastfeed in the high[er] degree research room for my school. It's all women, they're all lovely people and all friends of mine and so they were really supportive of me feeding or pumping. [IV 6]

I have a wonderful and supportive group of colleagues and friends who made me feel safe breastfeeding my baby in our shared office space. [OLS]

The importance of these supportive relationships is illustrated in the following account from one woman, who stated, “I’m kind of conscious of the fact that maybe that’s kind of...person-specific, and it’s more about the relationships rather than the policies or structures around that.”

For women who did not have this support, breastfeeding was a great challenge. For instance, several women spoke about their difficulty discussing breastfeeding with male supervisors:

With a male, or a different manager who I didn't know so well, it could have been quite stressful, but I guess those things happen when you're juggling returning to work and babies. [IV 9]

With my first child I remember coming into work for a half day meeting and feeling too embarrassed to ask my male supervisor for time during the day to express. My breasts were enormous and extremely painful by the end of the day and it was horrible sitting in the meeting and feeling the let-down reflex and hoping that I didn't start leaking through my shirt. The whole meeting was made up of men or women who hadn't had children and already I felt that my maternity leave from the project had been a hindrance so asking for 30 minutes to breastfeed was just more of me being a hassle. In the end when I returned to work full-time after 10 months maternity leave I decided it was easier to wean than to try and maintain breastfeeding. [OLS]

#### Importance of proactive support for breastfeeding

Supervisors who were proactive in providing support were most valued by women. These individuals initiated conversations with the breastfeeding woman, which lessened the apprehension that the woman might feel in requesting certain additional or special requirements. For instance, one women, who had been a student and a staff member at the university, spoke about a conversation with her postgraduate supervisor about breastfeeding when she returned to campus, saying, “she probably initiated the conversation, which just made all the difference because I just felt completely at ease and comfortable”. For women who did not have openly supportive supervisors or managers, they had to take a “proactive role” in initiating conversations about breastfeeding. Once women had raised the issue of breastfeeding, supervisors and managers tended to be supportive, as the following woman’s quote shows:

I didn't know where I could pump, and I didn't really ask my supervisor about a great deal, but when I did sort of broach the topic with him he said, ‘Oh, you can use any room you like’.

Women also spoke about the importance of ongoing education for managers and other workplace employees, to increase awareness about the importance of breastfeeding, and to take the onus off women to generate the support they need to breastfeed. For example, the following participant said, “It would be good if those in supervisory roles were made aware of these needs and opportunities and actively supported them — instead of requiring mothers to take the proactive role in requesting them.” The desire for greater understanding for women breastfeeding on campus was shared by undergraduate students, as the following quote shows: “Tutors could be more understanding/aware of special needs or requirements like breastfeeding. I have never felt comfortable discussing it with my tutors.”

### Theme two: finding private and safe spaces for breastfeeding

Staff and students at the university spoke about the importance of having a private space in which to breastfeed and express milk. For instance, women spoke about the need for internal locking doors in order for them to feel safe while breastfeeding and expressing. The need for privacy was particularly important when it came to expressing breast milk, as the following account demonstrates:

There’s something about expressing that you feel like . . . if somebody walks in on you breastfeeding, you feel like I'm with my child [but] there's something quite mechanical about expressing. I don't think it's as socially acceptable. It's not. It's a really private act. [IV 9]

Another component of feeling safe for some of the women in this study, was being able to avoid being seen by men, either whilst breastfeeding or expressing breast milk. Whilst participants applauded the university for having parenting rooms, many felt that co-locating breastfeeding facilities in spaces where men, with children, could also enter, deterred women from using that space for breastfeeding. This was most acutely felt by women who had a cultural or religious expectation to be fully covered when in the company of men. These experiences are captured in the following accounts:

It was getting annoying that the parents room was open to fathers which I understand but there's not enough privacy for a woman to breastfeed as I am a scarved Muslim woman and don't expose my body to anyone. [OLS]

I don't want a male watching me breastfeed . . . a parent’s room should be available for dads and mums with a separate private breastfeeding room. [OLS]

I use the Muslim female prayer room as I know definitely no men will be walking by. [OLS]

#### Assessing designated breastfeeding spaces on campus

Many women were unaware that there were parenting and women’s rooms on most of the university’s campuses advertised as spaces designated for breastfeeding or expressing. For instance, a woman said, “on campus there is no place for breastfeeding, no place to store pumped milk”. A staff member, who had also been a student at the university made the following comment:

As a student, I'm not sure where you would access this kind of information about parenting facilities or your rights in relation to breastfeeding during class. I don't recall ever seeing anything that was publicly available. [IV7]

Among staff and students who were aware of the facilities available to breastfeeding women, a number reported difficulties locating or accessing the spaces, as the following quotes illustrate: “The sign is small and easy to walk past, [and] not realise it is there.”; or “The rooms have always been closed when I needed them, then I’d needed to make my way to [another] block. This task becomes very difficult with a crying, hungry baby.” The difficulty of navigating the university campus while breastfeeding was discussed by a woman who was a staff member and student at the university, who commented:

I wouldn't even want to think about how hard it is for students who don't have their own offices, and then trying to negotiate their books and their baby. It must be really frustrating. [IV 6]

This situation was the same for staff visiting different campuses. One staff member who was at another campus for a work function disclosed her frustration in trying to navigate the environment without easy access to her office:

… there’s that feeling, I'm running around a campus to put a machine on my breast when my baby is so far away and really I want to be home feeding my baby right now. In that particular instance, it was the third room I'd gone to and it was locked, I was like, Can you just give me access? [IV 9].

Most women who were able to access these spaces were dissatisfied with the facilities, describing them as “small and not very private”, and lacking features such as power plugs, a sink or fridge. This experience was described by a student and casual academic staff member in the following account:

I expressed milk in the breastfeeding room which is joined to the disability room. I barely noticed it existed so was lucky to find it. Students go in there [for example able-bodied students go into the disability space] which was disruptive. One staff member yelled at me because I didn’t look disabled when I was in there. I showed her my milk and she apologised. There was no easy power point access near the chair and no nearby sink either. Both are really needed for expressing! [OLS]

#### Car parks, corridors, and classrooms

A lack of awareness about available facilities, an inability to access, or a dissatisfaction with designated spaces, led staff and students to breastfeed in other parts of the campus, such as disabled toilets, staff rooms, and outdoor areas. Some women were happy to breastfeed “anywhere”, and many students who reported breastfeeding in class reported that they had “no issues”.

However, other women found it challenging to find a space on campus where they felt comfortable to breastfeed. Consequently, these women had to make use of alternative spaces, as the following accounts show:

I could not find private spaces. I have expressed in my car on hot days. I have found it very stressful. [OLS]

I breastfed my young son on campus multiple times. I sometimes struggled to find a quiet, sheltered place to breastfeed that wasn't a corridor. [OLS]

[I used a] disabled toilet, and empty office — [it was] very demoralising. [OLS]

My experience was horrible, I had to use a disabled toilet. [OLS]

I asked for a suitable expressing room on my campus and was offered a large bathroom where I could sit on the side of the bath, or the first aid room — which could be entered at any time by any staff member and provided no real privacy. [OLS]

Some students reported accessing the designated breastfeeding spaces only to find them locked during regular business hours.

#### Having access to an office

Having access to a private office created a positive work environment for breastfeeding women, enabling the expression of milk or breastfeeding. Those women who had access to a private office were often aware of the benefits of this, and commented: “I feel like really fortunate, because a lot of other women wouldn’t have that facility available to them” [professional staff member]. However, women were often unsure whether it was against the policy to feed, or express, in their office, and others went as far as to install their own small fridge in their office to store expressed milk. A professional staff member shared the following experience:

I was doing it in my office, which I don't know is against policy or not but everyone else was happy with it. I had my own office. It's not accessible without swipe cards, so it's not like students could get into here without someone letting them in here. I put up a sign on the door. I just said, “Please, do not enter”, or something like that. Everyone knew what that meant [IV 5]

Having an office had many advantages for women as they did not have to navigate their way to the breastfeeding rooms, as one academic staff member explained:

I have an office and I use my office for breastfeeding, rather than the breastfeeding rooms. There is a parent's room in my building … but I don't feel comfortable breastfeeding in there because it's a glass room. It actually was quite suitable to do it in my office because I was able to keep everything here that I needed and … I didn't have to transport the pump backwards and forwards. [IV 8]

#### Having a safe place to store breast milk

Safety was paramount when looking for options to store breast milk. The conditions needed for safe breast milk storage were described by a professional staff member, who said:

. . . it was safe in that space. It was accessible, I knew no one was going to go in and steal it or was going to mix it up with anyone else's because I was the only breastfeeding mum in that room. [IV 6]

All women reported difficulties in accessing safe places to store their breast milk, however, these difficulties were more prominent for students and casual staff members. Even when casual staff did have access to a fridge they were concerned about the high usage of the fridge, and, how safe it was to store their milk there. As one woman said:

A fridge to store my expressed milk would have been nice, however, given it is a public space I would feel hesitant to leave my milk in a public fridge that others have access to. [OLS]

Concerns about storing expressed milk in a shared fridge, which was also used for other people’s lunches, was also off-putting for some participants. This caused embarrassment for one professional staff member who said:

But then I had to store my breast milk in the fridge or freezer, where everybody's lunches are kept … There's no separate facility for any of that, and so I hid them in bags so that people didn't necessarily know what was going on because I felt a little bit embarrassed about having them in with food. [IV 3]

There were also concerns about the milk being contaminated. Participants described placing their expressed milk into boxes to separate it from other people’s food and discussed concerns about fridges being “busy” and breast milk “going off”. Dissatisfied with on-campus options, some staff drove home to express and store their milk and then returned to work. Others had to “work around the system” accessing personal fridges installed in offices, as a professional staff member explained:

The staff fridges are always really full and it's not necessarily always very hygienic … when you're storing something like breast milk and you're going to go put that in your baby’s mouth, it's imperative that it's kept as sterile as possible. Pretty much my experiences of breastfeeding have been about working around the system. [IV 6]

### Theme three: feeling self-conscious and unprofessional

Many staff and students spoke about feeling uncomfortable breastfeeding at university. Students responding to the online survey wrote, “I felt conscious of making other students/staff uncomfortable at times.” Staff members described feeling that breastfeeding was “unprofessional”. This made breastfeeding particularly risky for staff on casual contracts. A postgraduate student and casual academic explained, “ [breastfeeding] might make me look a bit unprofessional because I don’t have that protection as a full-time staff member because I’m casual.” A staff member in a permanent academic role said:

. . . when I'm not at work I don't mind breastfeeding anywhere. I mean, baby's hungry, I just breastfeed wherever I am, bus stop, café, wherever I am. I don't mind, but at work it's a bit different. For me it really feels different. [IV 8]

A woman who was a postgraduate student and casual academic staff member spoke about being self-conscious breastfeeding in front of students she taught:

I was self-conscious about the fact that I had my top down and was feeding my baby … I felt conscious of the fact that, you know, I'm an academic and I shouldn't necessarily be feeding while teaching a class, but that was my own feelings, nothing that was said to me by anyone else. [IV 4]

She also spoke about feeling uncomfortable breastfeeding around colleagues: “I ended up pumping in my car instead of in the lunchroom just because I wasn’t very comfortable with how it might be making other people feel”. Wearing professional work clothes also served as a reminder to women that they were in the professional sphere now and that breastfeeding may not be compatible with that sphere. A professional staff member spoke about trying to express milk at work saying, “I’m in my professional work clothes where they’re not...like they’re not breastfeeding clothes, and so I’d have to undress half way down.”.

Washing up breast expression equipment offered another opportunity for women to feel some level of shame that they were combining work with breastfeeding. A professional staff member at the university said:

I always felt really weird standing there in front of someone with my breast pump rinsing it out. What I would do is rinse it out and just let it air dry, but then sterilize it at home every night. That was just my personal awkward thing because I don't know, it was some sort of visual thing that I thought people were seeing the pump that sits on my breast. It just felt funny. No one ever said anything. No one ever made me feel uncomfortable about it. [IV 5]

This clash of the public and private led some women to try to avoid being detected as a breastfeeding mother. A professional staff member shared the following experience:

I definitely felt stealth. I'd put it in a plastic bag and I'd shuffle down to the toilet. Luckily, my office as well, it's like single cubicle. I'd go in and lock the door and rinse it out there. If I went down to the toilet and it was occupied, for example, I'd have to carry this bag back to my desk and kind of have it under the desk till I had an opportunity. I guess it's any kind of mixing your professional and your personal life**,** it's going to be a little bit awkward. [IV 9]

Whilst participants acknowledged that no one deliberately made them feel uncomfortable, the lack of a supportive, visible and accepted breastfeeding culture made participants feel that they were behaving inappropriately.

#### Doing the wrong thing

Feelings of guilt about taking time out to breastfeed were also a feature of women’s experiences of returning to work at university. Designated lactation breaks were available to staff, however some women felt that these were perceived negatively by other staff members, as illustrated in the following quotes:

When you’re breastfeeding, you feel like you're doing the wrong thing. When you're at work, you feel like you need to be working and you feel guilty that you're taking time away to do something that is kind of a break. [IV 5]

Some staff held resentment towards me for taking [lactation] breaks. [OLS]

To overcome perceived attitudes about unprofessional behaviour women tried to enhance their outputs by combining the time spent expressing milk with completing work tasks, either by doing these simultaneously, or by staying back at the end of the day. Women made the following comments:

I actually used to take my computer into the room and work at the same time, because I had a hands-free pump and would literally do my emails and things at that same time. It turned out, I ended up doing that same habit [using pump while working] here and I couldn't produce enough milk. My milk supply slowed up and I couldn't feed anymore for that lunchtime feed that I was doing. It was difficult, but I think it's just one of those things. [IV 5]

Even though they say that you can have a lactation break, the work load doesn't reduce and you're expected to answer the phone or be on emails . . . I would stay back to finish a task or something or work from home to finish off what I needed to do. Because if you don't give it 110% like everybody else. . . you're letting the team down. [IV 3]

Another woman said, “It is hard to ask for these things as you already feel like a burden on the university by taking maternity leave and the limitations you have as a mother when you return to work.”

### Theme four: developing resilience to judgement

Several women spoke about the difficulty of breastfeeding at university, given that breastfeeding is still an unusual practice to observe in the public sphere. Women understood that they had the right to breastfeed but felt that it was still not an acceptable and normal thing for a woman to do on campus. For example, one woman said:

I understand that, legally, I have the right to breastfeed or have the right to a safe space to express breast milk, I understand all the legal considerations, but I can't help but feel, at times, that, you know, breastfeeding has not become the societal norm yet. And so, I do feel that even though nobody has directly judged me, I do feel like there would be judgment there. [IV 4]

Another woman shared this feeling, saying, “because it’s not normalised, because not everybody is doing it on campus, you do feel like a bit of an outsider...”. A postgraduate student and casual academic explained how breastfeeding at university required you to develop a “thick skin”:

I’m also at the stage now where I'm sort of like I have a much tougher skin and I’m not concerned about what other people are thinking about me pumping breast milk and washing things in sinks, and all of that. [IV 4]

Feeding a hungry baby wherever and whenever they needed became the priority, as did the desire to increase the visibility of breastfeeding in order to shift public attitudes to one of acceptance. A professional staff member said, “It was a bit embarrassing and nerve-wracking at first, but … once you overcome it, it was fine. It’s just what I did, and people didn’t seem phased by it at all”. A postgraduate student and professional staff member said:

The first time around I was really anxious, and because I wasn't comfortable and confident with breastfeeding, I'd want to breastfeed in private. But the second time around, I was much more confident to breastfeed in public and my perceptions of what people thought . . . I really didn't care, I was breastfeeding, my daughter was hungry, it was just nothing wrong with breastfeeding in public. [IV 7]

The above quote highlights how subsequent experiences with breastfeeding helped to build women’s confidence with breastfeeding at university. This confidence allowed women to become an advocate and role model for other women. An academic staff member who breastfed in front of students in class, described the event:

Hopefully, I had a bit of an impact also, educating, because most of my students are girls . . . being exposed to that model of combining breastfeeding and work. So, hopefully, it educated and inspired some of them. [IV 1]

## Discussion

This study has demonstrated that, even in an educational institution that prides itself on gender equity, and has policy provisions for parents in place, existing barriers can prevent women from reaching their breastfeeding goals and experiencing a supportive return to work or study. For staff we found that employment status had a significant impact on whether the university environment was supportive of breastfeeding. For casual staff and students, the environment was often difficult to navigate and many depended on the good will of others to enable their ongoing breastfeeding. The divergent experiences of these women, who traversed the university campus, revealed that their level of autonomy affected their experiences of combining breastfeeding and work or study.

Navigation of the space while following health department recommendations, by breastfeeding exclusively, and focusing on work or study while at university, created a dilemma for breastfeeding women. Barriers and obstacles deterred some women from continuing their breastfeeding relationship with their infant. This study provides insight into facilitators for ongoing breastfeeding on university campuses and how to create supportive and enabling breastfeeding environments for all.

### The balancing act — good mother vs good worker or student

The conflicting discourses of the good mother and the good worker are difficult for women to reconcile when combining return to work, or study, with breastfeeding [24, 25]. This period resembles walking on a tight rope between doing the right thing by one’s child and doing the right thing by one’s employer. Whist good mothers maintain their milk supply by expressing breast milk when separated from their child, good workers devote their time to getting work done with minimal interruptions [26]. This leads to what Gatrell has described as women becoming “boundary creatures, inhabiting ambiguously the idealized space between health definitions of ‘good’ mothering and organizational interpretations of what constitutes a ‘good’ employee” ([26] p. 6). Similar experiences are described by students who describe “… the internal struggle of having to choose between education and breastfeeding their infants” ([10] p. 210).

Breastfeeding bodies challenge the separation of the public-professional realm and the private-mothering realm [24]. Clashes between the merging of work and maternity have been debated for several years [27]. Breastfeeding at work has been deemed to be detrimental to a woman’s identity as a committed worker [25, 28]. There is an unspoken etiquette that assumes that women will conduct themselves in such a way as to keep breastfeeding, or the expression of breast milk, hidden from view and discreet [24, 25]. Women endeavour to keep breast expression out of view, with one study participant disclosing that a supervisor warned them that if they needed to express so often they should be at home with their infant full-time: “If you are at work then you are at work. You can’t expect people to put up with it” ([26] p. 15). Despite the availability of private offices, or designated spaces, participants in our study felt that there was an expectation that home life and work life should be kept separate. This attitude may reflect generational variations, especially in the context of historical trends in western societies, where women used to return to the workplace only after they had completed their child rearing [29].

Workplace gender equality and anti-discrimination legislation currently protects the rights of women with regard to breastfeeding and return to work or study [12, 13]. Whilst permanent university staff in this study reported feeling well supported to breastfeed when they returned to work, this was largely due to the maternity leave provisions. This meant that most returned when their infant was older, breastfeeding was well established and their infant was feeding less frequently. Employment and delayed return to work are known to have a positive effect on breastfeeding duration [30, 31].

Researchers have found that type of employment can make a difference to women’s experience of combining work and breastfeeding. Women in management and professional occupations have much more access to support when they return to work, when compared to women in temporary employment, or service industries, who report very low levels of support [32]. Women are hesitant to ask for adjustments to their schedule to enable breastfeeding for fear that it will be interpreted as a lack of commitment to the workplace and a privileging of their personal life [29].

Both staff and students in our study spoke about feeling self-conscious and embarrassed when expressing breast milk on campus. Some degree of feeling embarrassed was related to the possibility of men observing them breastfeeding or expressing milk. One study into English men’s perceptions of breastfeeding revealed a similar embarrassment experienced by men, many of whom viewed breasts as having a sexual function only. Men in the study felt conflicted between knowing it was a woman’s right to breastfeed but feeling that they would rather not see it in public [33]. These men also described embarrassment when even the word “breastfeeding” was mentioned. Focus groups with business managers in the United States revealed that using the term “breastfeeding” made some male supervisors feel uncomfortable and embarrassed. The focus group data also revealed a lack of knowledge about breastfeeding amongst this group of men, and fears about how hygienic this normal mode of feeding was [29]. Western cultural tolerance, and yet revulsion, towards public breastfeeding, has been captured in the following statement: “We want women to breastfeed, but we don’t want to see it — or even think about it happening” ([34] p.242). This dichotomous view contributes towards the feelings of embarrassment and discomfort that women describe.

Women report feeling self-conscious at work and that they are viewed as less professional, or uncommitted, if they combine breast milk expression with return to work [35]. Students on university campuses in North America revealed similar findings describing feeling embarrassed and self-conscious about expressing milk on campus [10, 36, 37]. Feeling self-conscious when breastfeeding has been shown to negatively impact on exclusive breastfeeding for the recommended duration [38].

In this study, permanent employees described feeling guilty for taking lactation breaks and conveyed a sense of letting the team down. Some described expressing milk whilst taking their lunch break, others stayed back after hours to make up for the paid lactation break, and some checked emails whilst expressing. This type of multi-tasking in order to maintain work outputs [29] can have a detrimental effect on breast milk supply, due to the impact of stress on the let-down reflex [19, 39]. Staff also reported feeling that they were doing the wrong thing if breastfeeding in front of students. Some authors describe this as, “Trying to juggle motherhood whilst trying to give the impression that motherhood isn’t affecting your ability to do your job” ([34] p. 173). Whilst this boundary crossing, between identities, is most acutely felt by staff, in other research students also described uneasiness when breastfeeding in class, or awkwardness when having to leave the class to attend to an infant in child care [10].

Support for women who are breastfeeding leads to improvements in their sense of contentment and wellbeing. Enhancement of personal life through workplace adjustments (such as being able to express milk] enhances employee satisfaction and wellbeing [40]. There are advantages for organisations and our society to support staff and students to fulfil their breastfeeding goals [4].

### The path of least resistance

One of the most common reasons for ceasing breastfeeding is return to work or study [5, 7–9, 11]. Having access to one’s own office, and having autonomy over the work schedule, facilitated many of our participants to continue breastfeeding. In this context navigation of a known landscape and access to a personal office enabled ongoing breastfeeding. Students and casual staff who had to navigate the wider university campus and an unknown landscape were dependent upon the organisation and the good will of individuals to enable their ongoing breastfeeding. Without access to acceptable safe spaces, students and casual staff either used their own car for a period of time, or stopped breastfeeding altogether.

The car represented what others have described as “my own private mobile space”, which is a last resort for many ([41] p. 10). Yet this space, which women retreat to, also symbolises the societal aversion to the breastfeeding body in public spaces. In an auto ethnographic account of breastfeeding in urban spaces, one author describes feeling “defeated by the abjection that my breasts, both their fluids and their social construction, produced” ([41] p. 10). The car represents a place of refuge for many as women describe being unable to find suitable places to express breast milk. Whilst the car offers some privacy, it is not an ideal space for breastfeeding, or expressing breast milk, as it can be cramped and very cold in winter and hot in summer [10].

The availability of suitable breastfeeding spaces clearly affects individual behaviour. A survey of higher education institutions in the United States revealed that the lowest number of breastfeeding rooms was 0.02 per 1000 students and the highest was 1.49 per 1000 students [42]. Some university campuses had a policy that placed the responsibility on the academic staff member to find a suitable space for students who are breastfeeding or expressing, when designated spaces were located too far away. Yet other campuses had ample spaces, access to lactation consultants and even provided breast pumps for student use [42]. Availability of these resources conveys to students that the university supports breastfeeding mothers and it transforms the environment into an enabling one.

Our results revealed that women often choose the path of least resistance when returning to work or study and breastfeeding. When ongoing breastfeeding is facilitated, women can combine their responsibilities at work or study with breastfeeding. However, if there are too many barriers and very few facilitators to ongoing breastfeeding, many will simply give up [17]. Women will choose to wean their infant upon return to work when support for continuing breastfeeding is absent or difficult to navigate [43]. Others have found that stressful negotiation of the childcare drop-off, combined with inability to access safe spaces for milk expression when separated from an infant, can also lead women to abandoning breastfeeding [10, 11, 42, 44].

A recent Cochrane review suggests that there is insufficient high quality evidence to evaluate the effectiveness of workplace support in improving breastfeeding duration rates [45]. Yet we know from breastfeeding survey data that one of the reasons provided by women for breastfeeding cessation is return to work [3]. It is clear that barriers to ongoing breastfeeding must be removed to enable women to reach their breastfeeding goals and create a more equal and just society.

### Creating a supportive environment for all

In a recent systematic review of employer-based programs to support mothers who are maintaining breastfeeding, the availability of spaces for breastfeeding or expression was the most common available support. The review included twenty-two articles across ten countries and included both public and private sector employers. The findings also revealed that the number of breastfeeding support items available directly correlated with the exclusive breastfeeding rate at 6 months, which indicated a dose-response effect. This study found that the more supportive initiatives available — such as access to a designated space with a fridge, and time to express or breastfeed — the more likely ongoing breastfeeding would be sustained [46].

There is a need to establish a parenting friendly community on university campuses to provide a way for breastfeeding students and staff to meet and support each other. Improving signage for breastfeeding spaces sends a message to the entire university community that breastfeeding is promoted and supported [10]. The organisational culture within which a woman breastfeeds clearly has an impact on breastfeeding rates. Being in an environment that has a named breastfeeding policy, for example, has been found to increase exclusive breastfeeding rates [18]. Receiving support for breastfeeding from co-workers has been shown to be an important factor in maintaining predominantly exclusive breastfeeding [47].

Unfortunately, the strategies women use to breastfeed according to imagined standards of discretion or privacy contributes to the marginalisation of breastfeeding in the workplace and the invisibility of women’s efforts in this area [25]. We argue, along with others, that improving breastfeeding rates is not simply the responsibility of individual women but of organisations and the broader society [1, 4]. Building enabling breastfeeding environments is not just about introducing supportive policies [29]; it is also about actively seeking to provide support for women who are returning to work or study. Awkward conversations with employers or educators about how to maintain the normal method of infant feeding whilst separated from one’s infant, is an indicator of a society that has not yet accepted breastfeeding as the norm. Proactive communication with breastfeeding women is required to facilitate ongoing breastfeeding [29]. Women are more likely to continue breastfeeding after return to work if they have been given information about organisational support for this prior to going on maternity leave [11]. We argue that workplaces should have breastfeeding policies, packages for women going on maternity leave and breastfeeding champions in the organisation who can advocate and support women when they return to work or study. Ultimately, organisations must accept that breastfeeding is the normal way to feed an infant and take all the necessary steps to become breastfeeding friendly environments [16]. This study has revealed how desperately this is needed, especially to support students and casual staff at universities who have access to the least amount of support and resources.

The economic benefit of promoting breastfeeding through the provision of mother-and baby-friendly work and school sites is unknown due to the lack of available research. We do know that retention of staff and students, and reduction in sick leave, increases satisfaction and feelings of loyalty, translating into cost savings for organisations [48]. We support the call to action to “Encourage educators to value breastfeeding as a public health issue and to protect, promote, and support breastfeeding” for students and staff ([48] p.435). We are happy to report that the higher education institution discussed in this paper, has committed to achieving the minimum standards set by the Australian Breastfeeding Association “Breastfeeding Friendly Workplace” Accreditation, for both staff and students, in response to these study findings.

## Conclusion and recommendations

This study has highlighted the experiences of staff and students returning to work or study at an Australian higher degree institution, whilst breastfeeding. The findings reveal that this educational institution, while aiming for gender equity, did not fulfil requirements for a breastfeeding friendly environment. Permanent employees of the university experienced a more supportive environment than did casual staff or students. Sustaining breastfeeding for the recommended duration, whilst working or studying, requires time and commitment on behalf of the mother, as well as a supportive workplace or study environment. Much needs to be done to ensure that university campuses are capable of supporting women to maintain breastfeeding and fulfil their desire to meet the optimal nutritional needs of their child.

### Limitations

This study was conducted at one university setting in NSW, Australia, and the results may not apply to all contexts. This study involved an online survey and interviews with individuals who self-nominated to be interviewed. The participating cohort may have become involved in the study because they had particular experiences to share which may not be reflective of all of the staff and student experiences.

### Recommendations

1. All organisations who employ women should have a designated breastfeeding policy.
2. Organisations should review their current provisions for breastfeeding women to ensure that they can comply with the minimum “Breastfeeding Friendly Workplace” criteria.
3. Information should be available at universities to ensure educators and managers are aware of the importance of breastfeeding, and the organisational commitment to supporting breastfeeding staff and students.
4. Educators and managers should be encouraged to be proactive when communicating support to breastfeeding women.
5. Organisations should cultivate and facilitate “breastfeeding champions” who can advocate, and provide peer support, to women returning to work or study,
6. Organisations should build supportive parenting communities to encourage greater acceptance and foster breastfeeding friendly environments.

## Abbreviations

IV: Interview
NSW: New South Wales
OLS: Online Survey
